# Antigen B modulates anti‐inflammatory cytokines in the EAE model of multiple sclerosis

**DOI:** 10.1002/brb3.2874

**Published:** 2022-12-29

**Authors:** Aliakbar Mariki, Zahra Barzin, Majid Fasihi Harandi, Kimia Karbasi Ravari, Mahboubeh Davoodi, Seyed Mohammad Mousavi, Soheila Rezakhani, Masoud Nazeri, Mohammad Shabani

**Affiliations:** ^1^ Student Research Committee Jiroft University of Medical Sciences Jiroft Iran; ^2^ Department of Parasitology and Mycology, School of Medicine Jiroft University of Medical Science Jiroft Kerman Iran; ^3^ Research Center for Hydatid Disease in Iran, School of Medicine Kerman University of Medical Sciences Kerman Iran; ^4^ Student Research Committee Yasuj University of Medical sciences Yasuj Iran; ^5^ Neuroscience Research Center, Neuropharmacology Institute Kerman University of Medical Sciences Kerman Iran; ^6^ Department of Anesthesiology Friedrich‐Alexander‐University Erlangen‐Nuremberg, University Hospital Erlangen Krankenhausstraße Germany

**Keywords:** Antigen B, cytokines, EAE, multiple sclerosis

## Abstract

**Introduction:**

: Multiple sclerosis (MS) is characterized by the destruction of the blood–brain barrier, loss of myelin sheath, and contribution of inflammatory interleukins such as TNF‐alpha, interleukin‐17, and interleukin‐6.

**Methods:**

: The current study investigated the effect of antigen B of hydatid cyst fluid on the reduction of anti‐inflammatory cytokines and nerve conduction velocity in rats with experimental autoimmune encephalomyelitis (EAE)‐induced MS. After isolation of antigen B from sterile cyst fluid, the rats were randomly divided into four groups: saline, EAE, EAE + teriflunomide (EAE + TF), and EAE + antigen B (EAE + AngB). The EAE model was induced using cow spinal cord homogenization, in combination with Freund's complete adjuvant. The serum concentration of cytokines including IL‐1B and IL‐17, IL‐10, IL‐6, and TNF‐X was measured by the ELISA method, and real‐time PCR was performed to study gene expression. Electrophysiological, behavioral, and neuropathological tests were also conducted.

**Results:**

: Nerve conduction velocity and IL‐10 concentration were increased in the antigen B group. The results of this study showed that antigen B reduced the inflammatory component of the EAE MS animal model by modulating the immune system compared to teriflunomide, which eventually led to a reduction in symptoms at the behavioral and electrophysiological level.

**Conclusions:**

: It seems that antigen B plays a critical role in regulating immunity and it can be used as a possible therapeutic agent to modulate the immune system in MS patients. It might be rational to consider hydatid cyst fluid antigen as a modifier in MS.

## 1 INTRODUCTION

Multiple sclerosis (MS) is a multifactorial, autoimmune, and inflammatory disorder in the central nervous system (CNS) (Koriem, 2016; Moosazadeh et al., 2021). The etiology of MS is unknown, and it is thought that a combination of genetic and environmental factors contributes to the MS pathophysiology. The role of different immune system factors in the emergence of MS symptoms has been investigated in different studies (Etemadifar et al., 2013; Hollenbach & Oksenberg, 2015; Karussis, 2014). The strongest genetic links with MS are human leukocyte antigens) HLA) including the alleles HLA‐DRB1*15:01, DQB1*03:02, and DQB1*03:01 (Didonna & Oksenberg, 2017). A spontaneous activity of T cell and B cell against central nervous system myelin has been found in the MS (Goldenberg, 2012; Hauser, 2015), and myelin protein components (including myelin basic protein, myelin‐associated glycoprotein, proteolipid protein, and myelin oligodendrocyte glycoprotein) are targeted by the immune system during different phases of the disease (Lemus et al., 2018). Microglia and macrophages living in the CNS are also involved in phagocytosis, antigen presentation, nerve demyelination, and cytokine production (Bogie et al., 2014; Voet et al., 2019). Th1‐cell cytokines activate macrophages, and activated macrophages destroy myelin and damage oligodendrocytes and they can produce other inflammatory cytokines that can aggravate nerve tissue damage (Rawji & Yong, 2013). Activated macrophages and microglia cells secrete a variety of cytokines such as TNF‐α, IL‐23, IL‐12, IL‐6, IL‐1, and IL‐17 that might damage oligodendrocytes and neurons (Cherry et al., 2014; James et al., 2020). Experimental autoimmune encephalomyelitis (EAE), as an animal model of MS, is induced by Th1‐cell immune responses, while the immune responses of Th2 cells produce IL‐4 and IL‐10 interleukins that could prevent EAE (Brambilla, 2019; Constantinescu et al., 2011; Danikowski et al., 2017). Any substance affecting the activity of Th1 and Th2 cells will probably be effective in inducing or preventing MS symptoms. Recent studies have demonstrated a modulatory effect for antigen B produced by the parasite *Echinococcus granulosus* on the immune system (Siracusano et al., 2008). Hydatid cyst occurs in the internal organs of intermediate hosts of *E. granulosus* including humans, and since the definitive hosts of this worm are dogs and canines, these animals infect intermediate hosts by passing fertilized eggs or worms through their feces. Antigen B in the hydatid cyst fluid produced by the parasite in humans causes humoral and cellular immune responses, and this process is orchestrated by simultaneous activity of Th1 and Th2 cytokines (Amri et al., 2008; Dakkak, 2010; Pourseif et al., 2018; Zhang et al., 2015). Macrophages are also involved in human hydatid cyst infection (Biswas & Mantovani, 2010). The parasite's strategies for escaping the immune system through antigen B are unique through intensifying Th2 activity (Rahimi et al., 2011) and reduction in the production of Th1 cytokines. In hydatidosis caused by this parasite, Th2 has a positive effect on the life of the parasite. In clinical infections with *E. granulosus*, the antigen B present in the fluid inside the cyst can act as an immunogen and guarantee the survival of the parasite in humans (Ait Aissa et al., 2006; Amri et al., 2007; Everts et al., 2010; Zhang et al., 2010, 2003). Evidence suggests that IL10 and IL4 attenuate the immune response of Th1 (Nono et al., 2012). Another way to modulate immunity with antigen B is to prevent the maturation of dendritic cells, which modulates the immune system in the individual.

Due to the fact that antigen B modulates immune responses, stimulates anti‐inflammatory responses, and converts inflammatory macrophages (M1) into anti‐inflammatory macrophages (M2) (Siracusano et al., 2009), in the current study, we investigated the anti‐inflammatory effects of antigen B on the EAE‐induced immune and electrophysiological alterations as an animal model of MS. Findings of the current study would provide more evidence for the immunomodulatory role of antigen B in MS.

## 2 MATERIALS AND METHOD

### 2.1 Animals

Male Wistar rats (n = 32, aged 6 weeks and weighing 220–240 gr) were purchased from Animal Farm of Kerman University of Medical Sciences. Rats were housed in the animals’ house of the Neuroscience center of Kerman University of Medical Sciences. Two rats were placed in each cage, and the room temperature was set at 23°C, with a 12/12 light/dark cycle. For adapting the rats to the room, they were brought to the study room 10 days before the onset of EAE induction procedure. Animals had free access to water and food ad libitum. On the 10th day, the rats were randomly divided into four groups: saline (healthy rats), EAE, EAE + triflunomide (EAE + TF), and EAE + antigen B. The study protocol was approved by the animal ethics committee of Kerman University of Medical Sciences (Code: IR.KMU.REC.1399.273).

### 2.2 EAE induction and drug administration

Spinal cord was extracted from cow, then mixed with distilled water, and homogenized with a homogenizer. The homogenized tissue was combined with equal amount (1:1) of Freund's complete adjuvant with a concentration of 10 mg/ml of *Mycobacterium tuberculosis*, and the resulting antigens were incubated for 24 h. In order to induce EAE model in the second, third, and fourth groups, 400 μl of the resulting antigen was injected subcutaneously into the waist of each rat. Immediately after injection of antigen and 48 h later, 100 μl of a combination of *Brutdellapara pretosis* with phosphate buffer saline at a concentration of half McFarland was injected intra peritoneally. Rat weights and degrees of muscle weakness were assessed daily. It took 12–14 days for the target groups to show EAE symptoms. Severity of disease from zero (no disease), one (tail disorder), two (tail paralysis), three (walking disorder), four (one‐leg paralysis), five (two‐leg paralysis), six (hands and feet paralysis), and seven (deaths) were recorded (Heidari Barchi Nezhad et al., 2019). Antigen B was administered orally to the fourth group every other day for seven times at a dosage of 1 mg, and the teriflunomide group also received 7 mg of teriflunomide (tebazio) orally per day. The control and EAE groups received 1 ml of drinking water orally per day to evaluate the effect of gavage.

### 2.3 Behavioral assessments

#### 2.3.1 Open field test

The open field test was used to investigate the possible effect of antigen B on motor disability and anxiety in rats with EAE induced MS. The apparatus consisted of a Plexiglass arena (90 × 90 × 45 [H] cm). Its floor was divided into central and peripheral regions, which contained 16 squares. At the beginning of each test, the rat was placed in the center of the square for 5 min. Then, the number of standing on the hind legs (rearing), rubbing the head and face with their toes (grooming), and the rate of velocity and mobility was recorded for each rat. At the end of each test, the square floor was cleaned with alcohol (Mohammadi et al., 2019).

#### 2.3.2 Rotarod test

The rotarod test was performed to check muscle strength and gait balance on the rod. Walking and running training on the rotating rod was given 1 day before the main test. Then, on the day of the test, the rat was placed on the rod with a changing speed rotation (10–60 RPM) three times. The interval between each test for each rat to rest was 30 min, and the time the rat fell was recorded by a chronometer (Mohammadi et al., 2019).

#### 2.3.3 Wire grip test

This hanging test was used to evaluate muscle function and disorders such as EAE‐induced imbalance in rats. The wire grip test included a horizontal steel wire (80 cm long, 7 mm in diameter) which was attached to two platforms on its sides. One day before the main test, rats were trained to hang from steel wire. Each rat was hung from the rod three times on the day of the test, and the rest interval between each test was 5 min for each rat. Time to fall for three tests was recorded; then, an average of three recorded numbers was taken as the endurance period of each rat (Mohammadi et al., 2019).

### 2.4 Electrophysiological evaluation

Wistar rats were anesthetized with intraperitoneal injection of ketamine and xylosine 31 days after induction of EAE. After shaving the back and top of the right hind leg of each rat, it was placed on a plate with a temperature of 35 ± 2 degrees so that it could better withstand anesthesia. Monopolar electrodes were placed subcutaneously on the right paw of the rat's hind leg to receive stimulation. Then, surface simulations were applied to the sciatic nerve in the upper part of the knee and on the ankle by bipolar needle electrodes. Motor nerve conduction velocity (MNCV) of the tibial sciatic nerve was recorded by the following formula (Power lab/ML856; AD Instruments, Sydney, NSW, Australia) (Zangiabadi et al., 2011):

$$\mathrm{NCV}=\frac{\mathrm{Distance}\;A(\mathrm{cm})-\mathrm{distance}\;B(\mathrm{cm})}{\mathrm{Onset}\;\mathrm{latency}\;\mathrm{notch}(s)-\mathrm{onset}\;\mathrm{latency}\;\mathrm{knee}(s)}$$

### 2.5 Cytokines measurement by ELISA reader

Thirty days after induction of EAE, spleen of rats was isolated. The spleen tissue was homogenized in saline phosphate and ripa buffers and then centrifuged at 30000 RPM. The obtained supernatant was used to measure cytokines. ELISA kits were purchased from Karmania Pars gene Company (Kerman, Iran) and then a brochure on the kits was used to measure the Intended cytokines. First, for standard preparation, three sterile 500 μl vials were prepared and added to all cavities and incubated for 25 min at room temperature. Then, 20 μl of the stopper solution was added to all cavities, and the adsorption rate of the samples was measured in an ELISA reader at 455 nm.

#### 2.5.1 Extraction of antigen B

A method devised by Oriol et al. was used to prepare and purify antigen B according to the additional instructions of Criag (Oriol et al., 1971). Hydatid cyst fluid (500 ml) was centrifuged at 30000×*g* for 10 min. This action resulted in the sediment of suspended particles such as protoscolexes. After removing the sediment, the supernatant was removed and poured into a dialysis bag and was dialyzed for 1 day in the presence of sodium acetate buffer 0.005 M with pH 5 and at a temperature of 4°C. This causes the main parasite antigens and globulins to become insoluble, but albumin remains soluble. After this step, the contents of the dialysis bag were centrifuged at 50,000×*g* with refrigerated ultracentrifuge for 50 min at vacuum and temperature of 4°C for 30 min, leading to precipitation of insoluble proteins including antigens 5 and B. The precipitate from the above step was mixed with 50 ml of 0.2 M phosphate buffer at pH 8, which causes the precipitate to dissolve. The above solution was saturated with ammonium sulfate to 40%, which removed globulins. For this purpose, while the solution of step 4 was gently stirred in a small container by a magnetic stirrer, a sufficient amount of ammonium sulfate powder was gradually dissolved in several steps. This operation took about 1 h. Then, the solution was centrifuged at 30000 *g* for 30 min. The supernatant was separated and placed in a boiling water bath (100°C for 15 min; Because antigen B is stable at high temperatures compared to other antigens, as a result, we can isolate this antigen by heating hydatid cyst fluid).  The above mixture was centrifuged by ultracentrifuge for 1 h. Antigen B dissolved in the supernatant was then collected and filtered through a Syringe head filter 0.2. Finally, 0.02% sodium azide (NaN3) was added. The antigen was stored at −70°C until use.

#### 2.5.2 Real time PCR

The spinal cord of rats was used to measure the expression of TLR4 genes. Note that 20–30mg of spinal cord tissue was homogenized in 500 μl of saline phosphate buffer and 500 μl of repa buffer. Then, a brochure on the kits of Karmania Pars Gene was used to measure gene expression. Relative quantification of the gene expression was carried out by Real‐Time Master mix with 5 μl SYBR Green PCR, 3 μl DEPC WATER, 1 μl activator and 1 μl extraction samples. Gene expression was measured in temperature profiles of 60°C, 30s and 95°C, 10s using Rotorgene device.

### 2.6 Fast blue luxury and hematoxylin and eosin staining

For neuropathological examination, rat spinal cord samples were isolated after 33 days. Then, it was placed in 10% formalin solution. The samples were first hydrated by 95% methanol. The samples were then kept in an alcohol–chloroform mixture for several hours. Samples were stored in fast blue luxury and hematoxylin and eosin (H&E) dye solution at 56°C. Excess dye was washed with 70% ethanol and distilled water. For differential staining, the slides were kept in lithium carbonate solution for 30 s and were then washed with distilled water, and at this stage, the samples were examined under a microscope. The gray and white materials were clearly identified, and the samples were kept in a Cresyl violet solution for 40 s and washed with distilled water. Samples were kept in absolute ethanol and xylene solutions twice for 5 min.

## 3 RESULTS

### 3.1 Body weight and neuropathological scores

**FIGURE 1:**
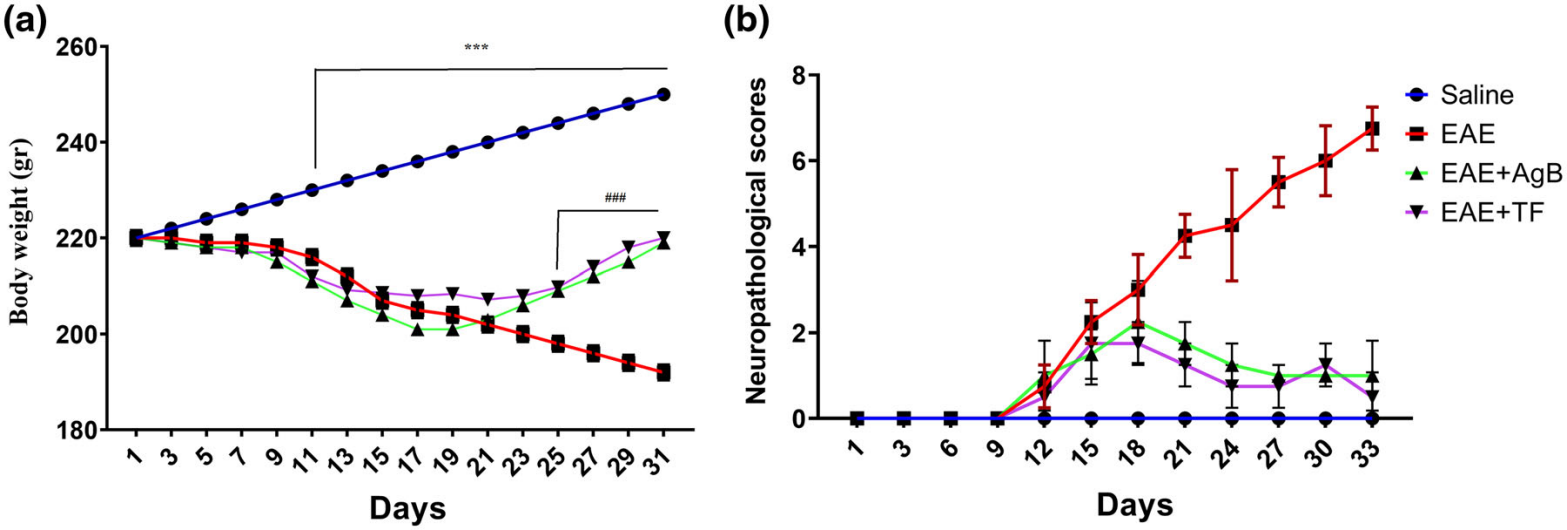
Effect of antigen B on body weight (a) and neurological score (b) during experimental autoimmune encephalomyelitis (EAE) induction and antigen B therapy compared with teriflunomide treatment. *In comparison to saline group, #in comparison to the EAE group, and +in comparison to EAE + AngB group. Results are shown as Mean ± SD.

In the EAE model, body weight and neuropathological scores indicate the severity and extent of the disease. The results showed that body weight in the EAE group was significantly reduced. Weight parameter in the EAE, EAE + AgB, and EAE + TF groups significantly decreased compared with the saline group. The results showed that body weight in EAE + AgB and EAE + TF groups on days 27–31 increased significantly compared with the EAE group (Figure 1a). The results of neuropathological examinations showed a progressing deterioration from the onset of induction in the EAE group. From day 12 onward, the EAE group had a progressing decline in neuropathological scores compared with the saline group and from day 22 onward compared with the EAE + AgB and EAE + TF groups. The results did not show a significant difference between the EAE + AgB and EAE + TF groups. The results also showed that antigen B and teriflunomide prevented the increase of disease severity from day 18, and the disease severity score was maintained (score 1) until day 33 (Figure 1b; *p* < .05).

### 3.2 Evaluation of locomotor activity using open field test

**FIGURE 2:**
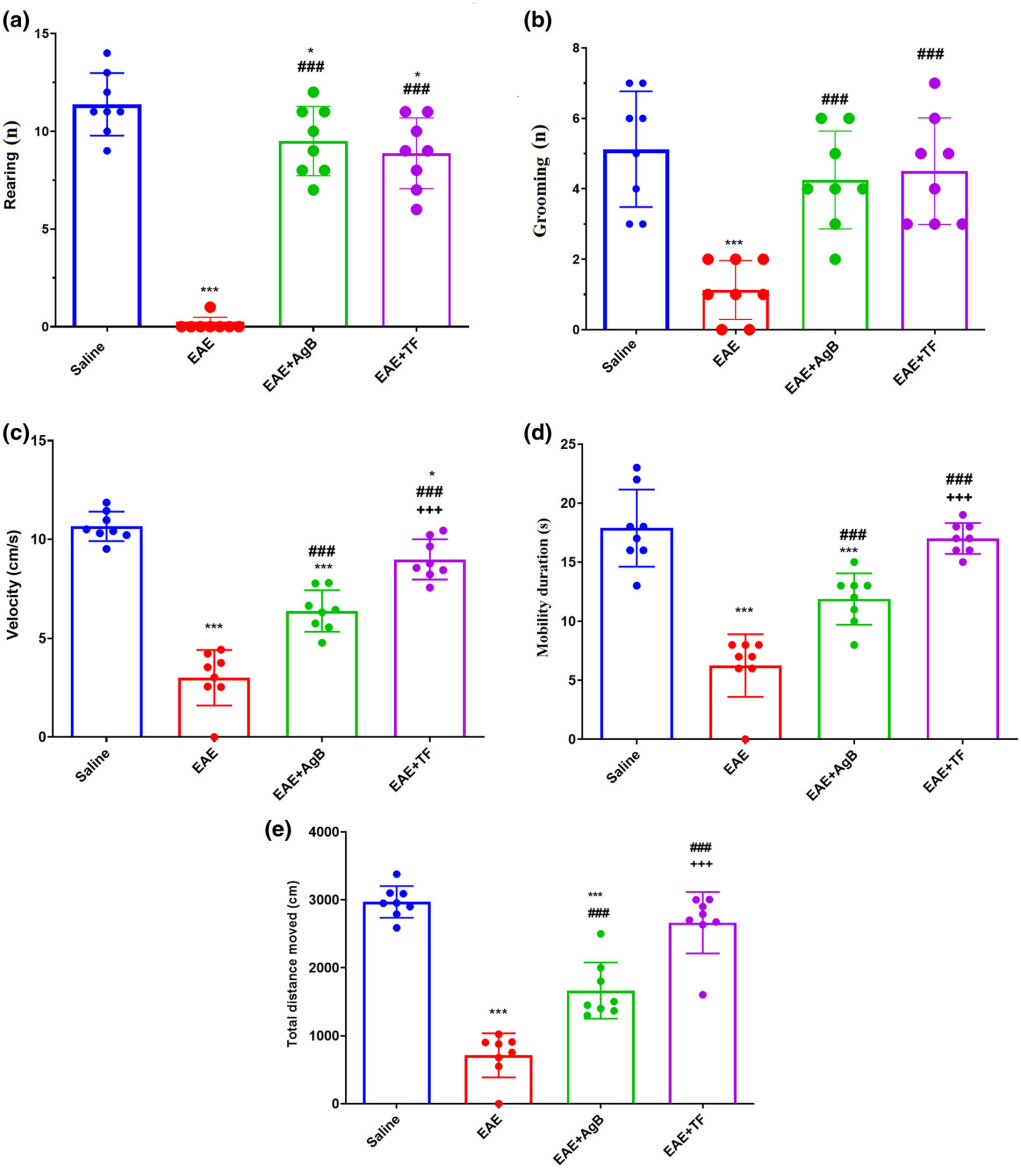
Effect of antigen B on locomotor activities after initiation of antigen B treatment compared with teriflunomide treatment. Symbols * and # significantly different from the saline and experimental autoimmune encephalomyelitis (EAE) groups, respectively. Results are shown as Mean ± SD.

**FIGURE 3:**
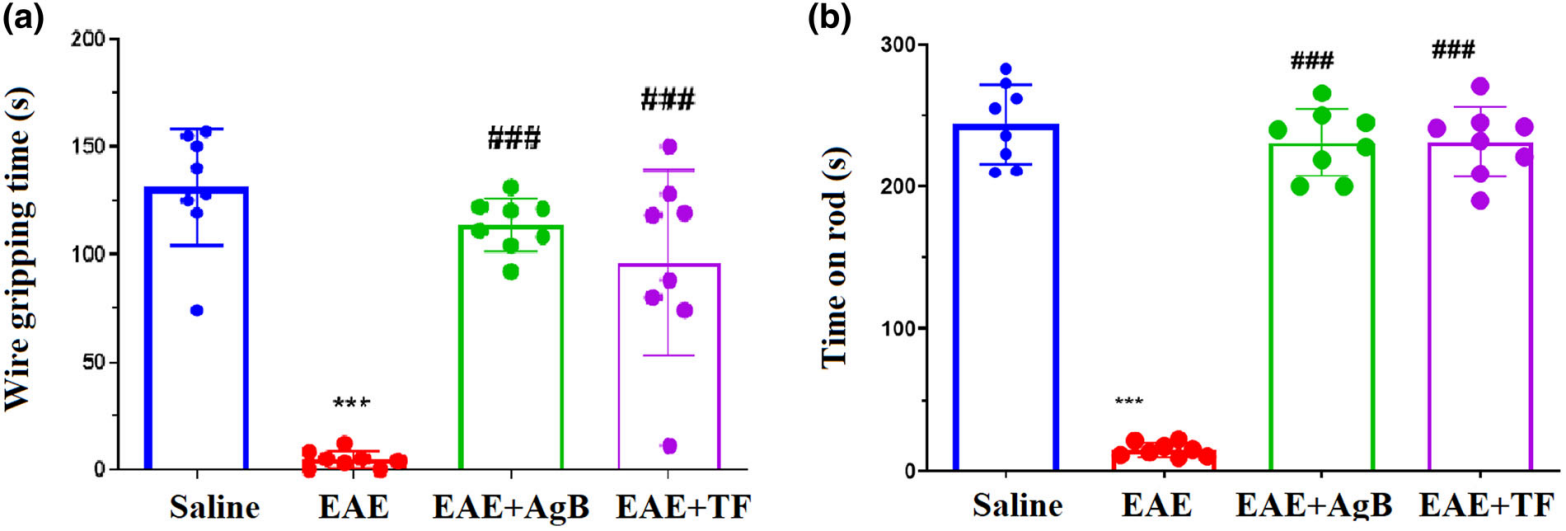
Effect of antigen B on muscle strength and muscle function after initiation of antigen B therapy compared with teriflunomide therapy. Symbols * and # significantly different from the saline and experimental autoimmune encephalomyelitis (EAE) groups, respectively. Results are shown as Mean ± SD.

Rearing analysis was performed in the open field test to evaluate the curiosity of rats in the four groups to stand on their hind legs. The results showed a significant reduction in the rearing number in the EAE‐treated groups compared with the saline group (*p* < .001 for EAE and *p* < .05 for EAE + AgB and EAE + TF). The results also showed a significant increase in the vertical activity of EAE + AgB and EAE + TF groups compared with the EAE group (*p* < .001, Figure 2a). The results showed a significant reduction in the number of grooming in the EAE group compared with the saline group (*p* < .001, Figure 2b). Evaluation of grooming number showed a significant increase in the EAE + TF and EAE + AgB groups compared with the EAE group (*p* < .001, Figure 3b). The results of mobility and velocity data were performed to evaluate the extent of stress‐related behaviors and to evaluate the effect of antigen B on the improvement of movement disorder induced by EAE induction. Mobility and velocity data as effects of antigen B on explorative and anxiety‐related behaviors showed that the amount of mobility time and velocity of rats in the EAE and EAE + AgB groups decreased compared with the saline group (Figure 2c,d; *p* < .001). These parameters in the EAE + AgB and EAE + TF groups had significantly increased compared with the EAE group, and similarly the mobility time and velocity in the EAE + TF group were higher compared with the EAE + AgB group (*p* < .05). In the open field test, EAE significantly decreased total distance moved (mean distance moved: *p* < .001). Total distance moved in the EAE + AgB and EAE + TF groups had significantly increased compared with the EAE group (Figure 2e; *p* < .001), and this parameter in the EAE + TF group was higher compared with the EAE + AgB group (*p* < .001).

### 3.3 Evaluation of hand muscle strength, balance, and walking strength

Wire grip test was used to assess the muscle strength of the four groups. The mean time of three consecutive experiments was recorded for each rat. Muscle strength in the EAE group was significantly reduced compared with the saline group (Figure 3a; *p* < .001). Our results showed that the EAE + AgB and EAE + TF groups could spend more time gripping the horizontal bar than the EAE group. Rotarod test was performed to evaluate locomotor strength and motor balance in the study groups. The results showed a significant reduction in gait strength and motor balance of the EAE group compared with the saline group (*p* < .001). The results showed an almost equal balance in the EAE + AgB group with the EAE + TF group. The results also showed a significant increase in time on the rod in the EAE + AgB and EAE + TF groups compared with the EAE group (Figure 3b; *p* < .001).

### 3.4 Motor nerve conduction velocity

**FIGURE 4:**
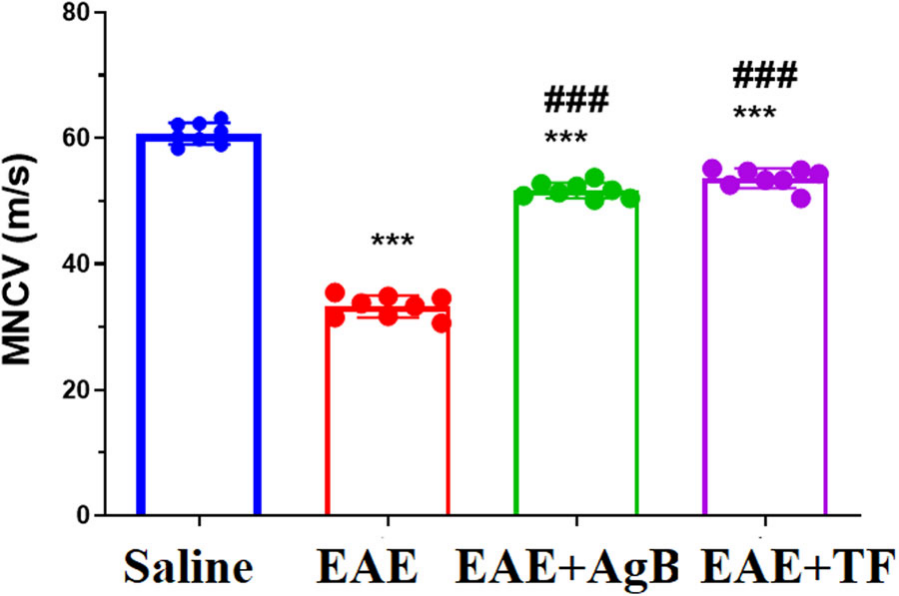
Effect of antigen B on nerve conduction velocity after initiation of antigen B treatment compared to teriflunomide treatment. Symbols * and # significantly different from the saline and experimental autoimmune encephalomyelitis (EAE) groups, respectively. Results are shown as Mean ± SD.

Electrical conduction velocity was measured in rats of the four groups 31 days after EAE induction. MNCV in the EAE (33.1 m/s), EAE + AgB (51.6 m/s), and EAE + TF groups (53.5 m/s) were significantly reduced (*p* < .001) compared with the saline group (60.7 m/s). Electrical conductivity was significantly increased in the EAE + TF and EAE + AgB groups compared to the EAE group) Figure 4; *p* < .001).

### 3.5 Evaluation of cytokines concentration by ELISA method

**FIGURE 5:**
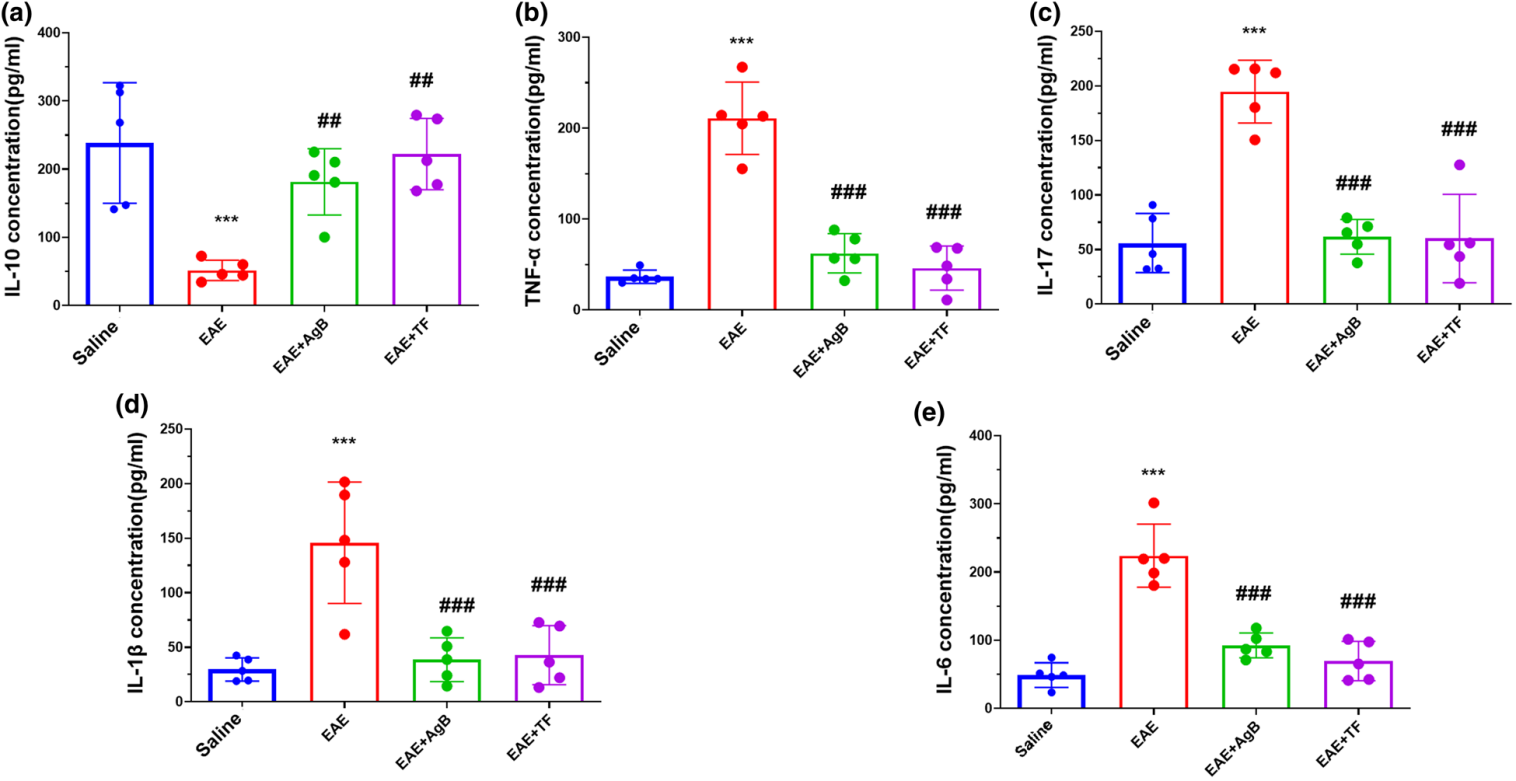
Effect of antigen B on the reduction of anti‐inflammatory cytokines IL‐10 (a) and inflammatory cytokines TNF‐α (b), IL‐17 (c), and IL‐1β (d) compared with teriflunomide treatment. Symbols * and # significantly different from the saline and experimental autoimmune encephalomyelitis (EAE) groups, respectively. Results are shown as Mean ± SD.

Five spleen samples from each group were randomly selected to measure interleukin 10. The concentration of interleukin 10 in the EAE group was significantly reduced compared with the saline group (Figure 5a; *p* < .001). Interleukin 10 in the EAE + TF and EAE + AgB groups was significantly increased compared with EAE group (*p* < .01). Concentrations of TNF‐α, IL‐17, IL‐1β, and IL‐6 cytokines were measured in the spleen samples of five rats from each study group to evaluate the extent of inflammation and the severity of nerve damage. The level of these inflammatory cytokines in the EAE group was significantly increased compared with the saline group) *p* < .001(. Concentration levels of cytokines TNF‐α, IL‐17, IL‐1β, and IL‐6 in the EAE + TF and EAE + AgB groups were significantly reduced compared with the EAE group (Figure 5a–e; *p* < .001).

### 3.6 Measurement of NOx and TLR‐4 mRNA level

**FIGURE 6:**
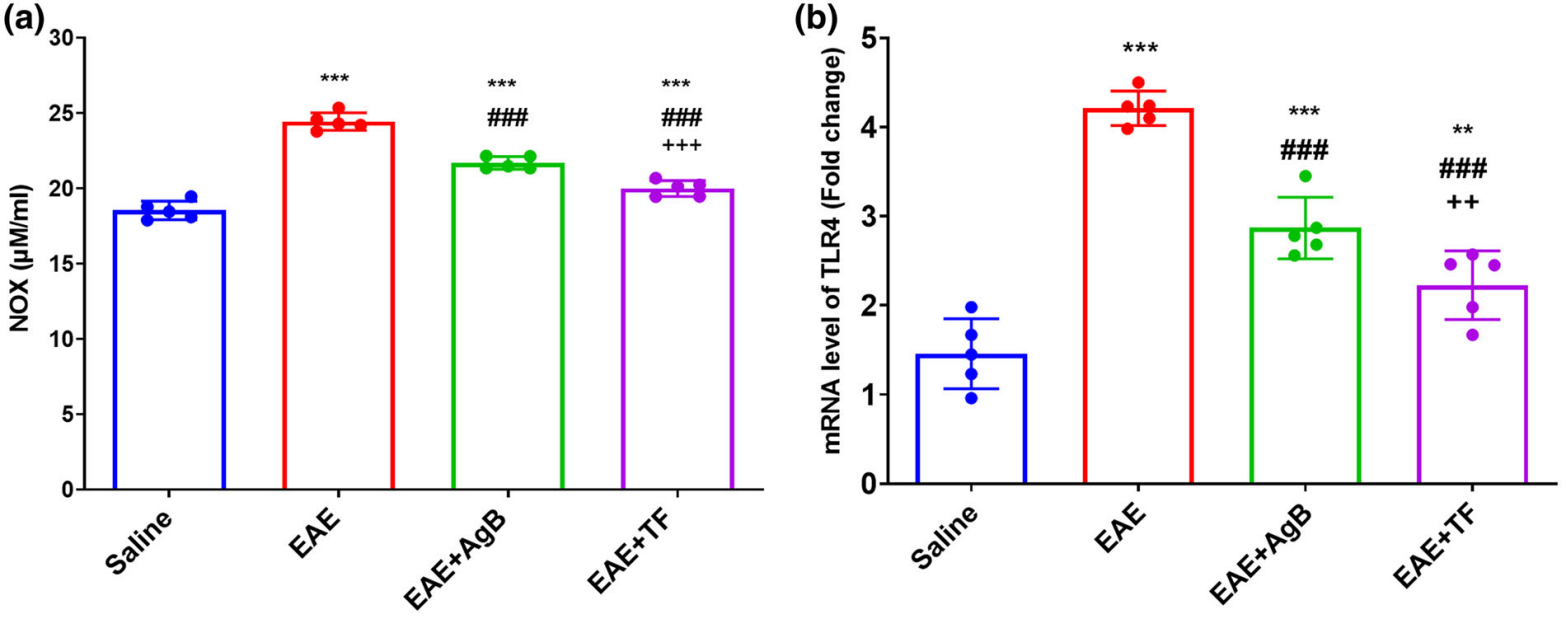
Effect of antigen B on the reduction of inflammatory factors IL‐6 (a), NO (b), and TLR4 (c) compared with teriflunomide treatment. Symbols *, #, and + significantly different from the saline, experimental autoimmune encephalomyelitis (EAE), and EAE + AgB groups, respectively. Results are shown as Mean ± SD.

Our data indicated that EAE was associated with higher serum NOx levels in the EAE group than levels seen in the saline and EAE + TF and EAE + AgB groups (Figure 6a; *p* < .001). Nitric oxide measurement is used to evaluate intracellular oxidative stress and apoptosis. The concentration of NOx in the EAE + TF group was significantly decreased compared with the EAE + AgB group (*p* < .001).

Data of TLR‐4 as one of the biomarkers of MS that plays a major role in the pathogenesis of this disease showed an increase in TLR‐4 gene expression in EAE rats (Figure 6b, EAE: 4.2 fold, *p* < .001; EAE + AgB: 2.86 fold, *p* < .01; EAE + TF: 2.22 fold, *p* < .01) compared with the saline group. There was also a significant decrease in the expression of TLR‐4 gene in the EAE + TF group (*p* < .01) compared with the EAE + AngB group.

### 3.7 Evaluation of demyelination rate and number of immune nucleated cells in spinal cord

**FIGURE 7:**
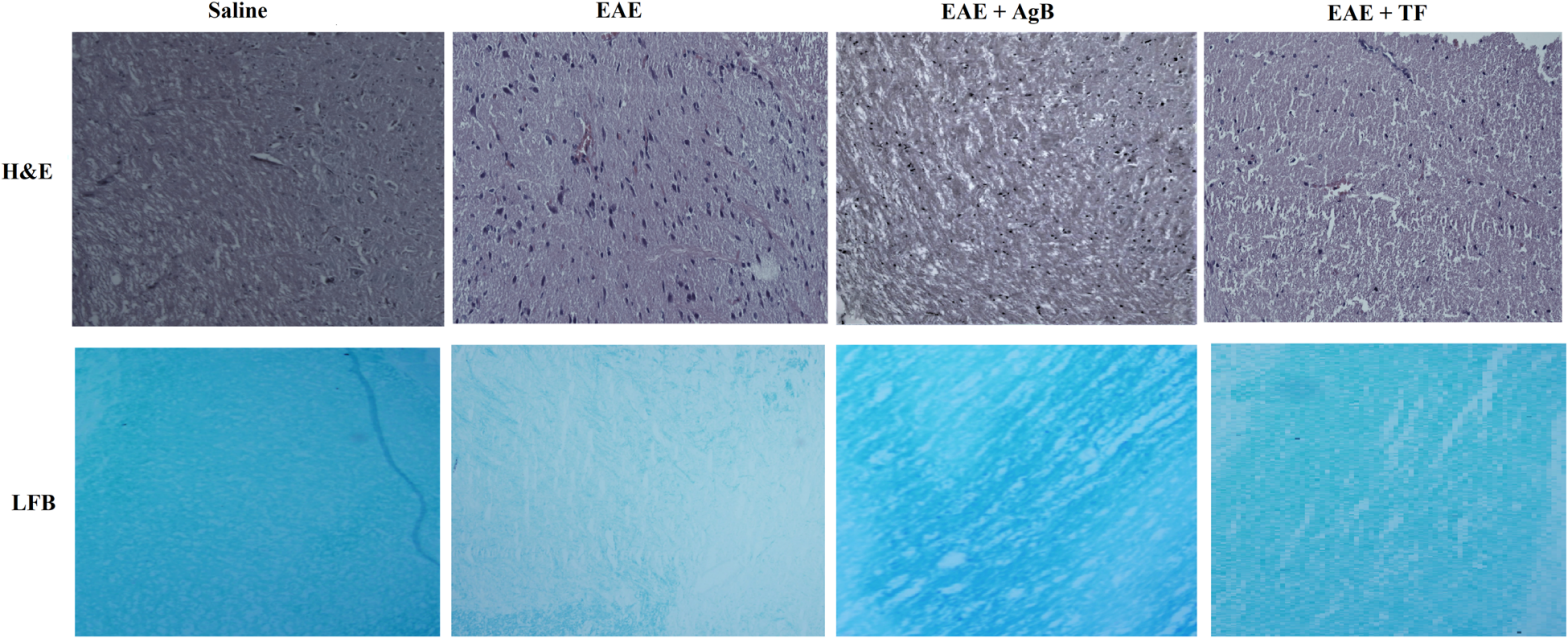
In this study, hematoxylin and eosin (H&E) staining of the cervical spinal cord was used to show the number of nucleated immune cells. The number of nucleated immune cells in the experimental autoimmune encephalomyelitis (EAE) group was significantly increased compared to the other study groups. In this study, Fast blue luxury staining was used to evaluate the extent of demyelination, and decreased color intensity indicates more demyelination, as observed in the EAE group.

Transverse examination of the cervical spinal cord section, which causes many sensory and motor disorders in MS, using H&E staining showed that the number of nucleated cells in the EAE group (44 per square micrometer) increased significantly compared with the other groups. While the number of nucleated cells in transverse section of antigen B group (18 per square micrometer) was higher than teriflunomide group (12 per square micrometer), no significant difference was found between the antigen B and teriflunomide groups. The number of nucleated cells in the saline group was 8 per square micrometer. Staining with Fast blue luxury, which is specific to the study of myelin, showed that the rate of demyelination in the EAE group was 89%, which was a significant increase compared with other groups. Rate of demyelination in antigen B, teriflunomide, and saline groups was 26%, 19%, and 0%, respectively (Figure 7).

## 4 DISCUSSION

The current study examined the effect of antigen B treatment on the animal model of MS and studied the concentration level of inflammatory cytokines which are known to play an important role in the pathogenesis of MS. Antigen B is demonstrated to modulate immune responses in different animal's models; thus, we studied its effect on the EAE‐induced behavioral, molecular, histological and NCV alterations with positive outcome. Results of this study provide evidence for beneficial effect of antigen B on symptoms of MS in an animal model, and future studies might use these data to provide modulatory treatments for these patients.

MS is a multi‐faceted disease with four main clinical types. In relapsing‐remitting MS (RRMS), inflammation could be a major cause of relapse (Baecher‐Allan et al., 2018). Drugs used in this type of MS are teriflunomide, interferon‐alpha, and interferon‐beta which reduce the number of relapses by modulating the immune system and improve patient recovery (Kamińska et al., 2017). However, these drugs are sometimes not effective in progressive forms, and more aggressive approaches are needed to control the progress of the disease. Studies have shown a greater degree of the blood–brain barrier impairment in the RRMS type (Fadda et al., 2021; Haile et al., 2017). There are several animal models for the study of multiple sclerosis, including EAE and cuprizone. This study aimed at investigating the effects of antigen B on the neuropathological scores and cytokines alterations in an EAE MS model.

EAE is the most common animal model for the study of MS, and the animal is immunized with a nerve antigen and inflammatory adjuvant which leads to the production of TH17 and TH1 that could impair the blood–brain barrier function and enter the brain and cause demyelination that can be either reversible or irreversible and has a relapsing‐remitting property like RRMS (Kipp et al., 2017). With regard to body weight and neurological scores, the initial symptoms began 9 days after the EAE induction and onset of the disease. As presented in results, weight loss was significant in all EAE groups 1 week after the model induction. Our results showed that antigen B alleviated weight loss, neurological deficits, and EAE disease progression. ELISA results, H&E staining, and Luxol fast blue of lumbar spinal cord cross sections confirmed that antigen B significantly reduced the demyelination and inflammation in the EAE animal model of MS.

In the early phase of EAE, as with MS, especially RRMS, glutamate transport is increased, leading to synaptopathy and an increased expression of inflammatory cytokines such as IL‐6, IL‐1B, and TNF‐X (Gatta et al., 2020; Millward et al., 2020; van der Star et al., 2012). Previous studies have shown that the mechanism of antigen B in modulating the immune system is the polarization of TH1 to TH2 that can reduce the production of inflammatory cytokines TH1 and TH17, and one of the mechanisms by which antigen B activates TH2 is its attachment to IgG4 (Ioppolo et al., 1996; Li et al., 2017). According to the above description, it is possible that the function of antigen B might be similar to teriflunomide and Interferon Beta‐1 as effective treatment modalities for RRMS than other various types of MS. This study is the first to investigate the effect of antigen B on reducing inflammation through the production of TH2 cytokines and its effect on nerve repair in the EAE model. The results of wire grasp and rotarod behavioral tests showed that antigen B, like teriflunomide, increased muscle strength, mobility, and balance in rats. Data also showed an increase in the curiosity of rats in the antigen B group toward their surroundings, almost like the saline group and also had less anxiety‐like behavior than the EAE group. ELISA results showed a strong effect of antigen B on the reduction of inflammatory cytokines such as IL‐1B, IL‐6, IL‐17, TNF‐X, and NOx, and IL‐10 was also increased. PCR real‐time results showed increased expression of TLR4 gene in the EAE group.

The MNCV test showed an increase in nerve conduction velocity in the antigen B group, which could be due to the effect of antigen B on the reduction of demyelination which was seen in a neuropathological study. Similar to our results, in the study of Soufli et al. which investigated the effect of soluble antigens on laminated layer of *E. granulosus* in inflammatory bowel disease in vivo in mice and showed that antigen B decreased the cytokines IL‐1ß, IL‐6, and TNF‐α by affecting the NF‐κB and IRAK pathways (Soufli et al., 2015).

In another similar study (Khelifi et al., 2017), the effect of the *E. granulosus* antigen B was investigated on NO and TNF‐X production pathways in ulcerative colitis animal model, and the activity of these pathways was diminished. Histopathological analysis of colon cells showed that cell death was significantly reduced in the antigen B‐treated group. Studies have shown that the AgB 12 kDa subunit is a potent protease inhibitor with the capacity of inhibiting polymorphonuclear cells (PMNs), especially neutrophils (Riganò et al., 2001). It can also regulate the synthesis of antigen B‐specific antibodies such as IgE and IgG4 (MacIntyre et al., 2000). Antigen B could also activate TH2 by binding to IgG4 and leads to the production of IL‐10, IL‐4, and IL‐13 and reduces IL‐6. IL‐4 and IL‐10 could have an anti‐inflammatory response by affecting monocytes (Y. Wang et al., 2015). In line with our results, the study of Rigano et al. (2007) showed that dendritic cells establish a strong link between acquired and innate immune cells. Dendritic cell's function depends on what pathogen they encounter with. Antigen B causes dendritic cells to stimulate the immune response to produce TH2 instead of TH1 and TH17 by inhibiting CD80 expression and increasing CD86 expression, and dendritic cells which become mature by AgB have a poor response to inflammatory and infectious stimuli. Consequently, dendritic cells produce interleukin‐10 and decrease the production of TNF‐X and NO (Abu‐Raddad et al., 2021). In contrast with the results of our study, the antigen B had no effect on changes in IL‐6 concentration in the study of Kanan and Chain (2006). Another study investigated the effect of the *E. granulosus* antigen B on airway inflammation and demonstrated that the increase in IL‐4 caused TH2 activation (H. Wang et al., 2014). IL‐4 is an important cytokine in the presence of antigen B for TH2 activation. The activation of TH2 increased IL‐10 and decreased IL‐5 and IL‐17A. IL‐17A is an important proinflammatory cytokine in the TH17 pathway, and its inhibition greatly suppresses the body's inflammatory system (Petrone et al., 2015). The antigen B stimulates macrophages to the M2 phenotype, which produces high levels of IL‐10, TGF‐β, and the enzyme arginase, as well as low levels of IL‐12 and IL‐23 which create an anti‐inflammatory status (Baz et al., 2006). Exposure to IL‐4 and IL‐13 activates immune complexes as well as M2 macrophages (Benveniste et al., 2004). Antigen B can also increase the number of FOXP3 proteins, also known as “Scurfin,” and eventually reduce the production of IL‐5 and IL‐17 by producing FOXP3 + regulatory T (Davis et al., 2013; Mejri et al., 2017; Silva‐Álvarez et al., 2016). In our study, the results showed that antigen B inactivates TH1, but Frazie et al. (2004) showed that antigen B has a double effect on both TH1 and TH2. It activates TH1, which in turn activates IL‐12 and increases IFN‐γ, leading to increased IL‐10. This study indicates that IL‐10 production is dependent on IL‐12 and IFN‐γ production, while our results demonstrated that IL‐6 production was not significantly altered in the AgB group. Future studies on the effect of antigen B on different IL subtypes seem to be necessary following different concentration of antigen B throughout different time windows.

## 5 CONCLUSION

The results of the present study showed that the severity of the disease in the experimental MS model increased during the study, but in the group receiving AgB, the symptoms reduced gradually over time. These findings indicate that antigen B demonstrated beneficial effects over a 30‐day period. It reduced both the severity of symptoms and the alteration of inflammatory and anti‐inflammatory cytokines. Furthermore, it increased myelin density and motor performance and decreased the level of NOx and TLR4. Therefore, it appears thatantigen B plays a critical role in regulating immunity and it can be used as a possible therapeutic agent to modulate the immune system in MS patients.

## AUTHOR CONTRIBUTIONS

Aliakbar Mariki and Zahra Barzin contributed to sample preparation, performed the experiments, and wrote the manuscript with input from all authors and also edited the manuscript. Majid Fasihi Harandi conceived of the presented idea and contributed to the interpretation of the results. Kimia Karbasi Ravari, Mahboubeh Davoodi, and Mohammad Mousavi consulted the behavioral and molecular tests. Soheila Rezakhani and Masoud Nazeri consulted the analytic calculations, pharmacological study and contributed to sample preparation. Mohammad Shabani supervised the project, contributed to the interpretation of the results, designed the experiments, and derived the models and analyzed the data.

## CONFLICT OF INTEREST

The authors declare no conflict of interest.

### PEER REVIEW

The peer review history for this article is available at https://publons.com/publon/10.1002/brb3.2874.

## Funding information

The authors gratefully acknowledge the Kerman Neuroscience Research Center and Jiroft Medical University for their support and assistance.

## Data Availability

The datasets used or analyzed during the current study are available from the corresponding author on reasonable request.
